# Effect of O-arm on reduction quality and functional recovery of acetabular dome impaction fractures: a retrospective clinical study

**DOI:** 10.1186/s12891-023-06987-6

**Published:** 2023-11-02

**Authors:** Hongli Deng, Yuxuan Cong, Jinlai Lei, Dongyang Li, Chao Ke, Zhiqiang Fan, Hu Wang, Pengfei Wang, Yan Zhuang

**Affiliations:** 1 Department of Orthopedic Trauma, Honghui Hospital, Xi’an Jiaotong University, No. 555, East Youyi Road, Xi’an, Shaanxi 710054 China; 2 Xi’an Medical University, No. 1, Xinwang Road, Weiyang District, Xi’an, Shaanxi 710021 China

**Keywords:** Acetabular dome impaction fracture, O-arm, Reduction quality, Functional recovery

## Abstract

**Background:**

Acetabular dome impaction fractures (ADIF) are difficult to reduce and have a high failure rate. Consistency between the acetabulum and the femoral head is usually assessed using intraoperative X-ray fluoroscopy to evaluate the quality of fracture reduction. This study examines the effects of intraoperative mobile 2D/3DX imaging system (O-arm) on the reduction quality and functional recovery of ADIF.

**Methods:**

We retrospectively analysed the data of 48 patients with ADIF treated at Honghui Hospital between October 2018 and October 2021.The patients were divided into the X-ray and O-arm groups. The residual step-off and gap displacements in the acetabular dome region were measured, and fracture reduction quality was evaluated. Hip function was evaluated using the modified Merle d’Aubigné and Postel scoring systems.

**Results:**

There were no significant intergroup differences in the preoperative general data (p > 0.05). The mean residual average step displacement in the acetabular dome region was 3.48 ± 2.43 mm and 1.61 ± 1.16 mm (p < 0.05), while the mean gap displacement was 6.72 ± 3.69 mm and 3.83 ± 1.67 mm (p < 0.05) in the X-ray and the O-arm groups, respectively. In the X-ray group, according to the fracture reduction criteria described by Verbeek and Moed et al., one case was excellent, 13 cases were good, 11 cases were poor; 56% were excellent or good. In the O-arm group, seven cases were excellent, 12 cases were good, and four cases were poor; overall in this group, 82.6% were excellent or good (p < 0.05). A total of 46 patients achieved fracture healing at the last follow-up. In the X-ray group, according to the modified Merle d’Aubigné and Postel function score, three cases were excellent,12 cases were good, six cases were middle, three cases were poor; 62.5% were excellent or good, In the O-arm group, 15 cases were excellent, four cases were good, two cases were middle, one case was poor; 86.4% were excellent or good (p < 0.05).

**Conclusions:**

The application of O-arm in ADIF can improve fracture reduction quality and functional recovery.

## Background

As the population ages, the incidence of acetabular fractures increases. Due to varying degrees of osteoporosis in older adults, low-energy injuries can lead to acetabular fractures. When a fracture involves the acetabular dome, there are usually different degrees of compression. Imaging usually shows that the acetabular dome has a typical double arc shadow, similar to the wings of a seagull in flight, which is called the “seagull sign”. Previous literature [1] has reported that acetabular fractures with the “seagull sign” are difficult to treat and usually indicate a poor prognosis. Therefore, in recent years, scholars have been devoted themselves to studying ADIF.

**Fig. 1:**
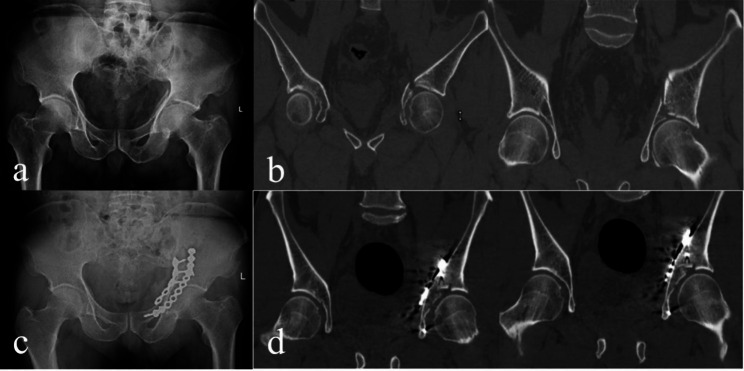
A male patient, aged 64, who presented with anterior column and posterior hemi-transverse fracture from a traffic accident. (**a, b**) Preoperative X-ray and CT scan revealed acetabular dome impaction fractures. (**c**) Postoperative X-ray showed satisfactory reduction of the fracture. (**d**) Postoperative CT showed 3.7 mm step displacement of the acetabular dome

Restoring good anatomical relationships is the key to functional recovery after ADIF. Residual steps or gaps in the acetabular dome after fracture reduction can increase the local contact pressure on the joint, leading to traumatic arthritis. Li et al. [2]showed that if the residual articular surface step of the acetabular dome exceeds 2 mm, the local maximum pressure can increase 50%, eventually leading to traumatic arthritis and significant impact on hip function. Schreurs et al. reported similar conclusions [3]. Therefore, it is particularly important to effectively assess fracture reduction quality and achieve as much anatomical reduction as possible. Currently, the evaluation of intraoperative reduction of ADIF is usually done under X-ray fluoroscopy [4–6]. However, the acetabulum has a spherical three-dimensional structure, and part of its articular surface cannot be accurately observed using X-ray fluoroscopy. To date, CT evaluation is usually performed after surgery for acetabular fractures [7, 8], and intraoperative use of CT to evaluate and guide fracture reduction is rarely reported. Conventional postoperative computed tomography (CT) reveals significant residual displacement in some layers of the acetabular dome (Fig. 1). It has been reported that when CT scans are routinely performed after acetabular fractures with satisfactory radiographic reduction assessment, 2.5% of patients require revision surgery [9].

With the advancement of medical technology, the O-arm has gradually demonstrated superiority in complex operations, such as spine and maxillofacial surgery, owing to its mobile completion of high-quality three-dimensional (3D) imaging and transmission [10–13]. However, to the best of our knowledge, no study to date has evaluated the advantages of the O-arm for ADIF treatment. The main purpose of this study was to explore the effect of the O-arm versus traditional X-ray fluoroscopy on the reduction quality and functional rehabilitation of ADIF and analyse its application value.

.

## Materials and methods

### Inclusion criteria and exclusion criteria

Inclusion criteria: ① fresh ADIF (< 3 weeks).② no osteoarthritis of the affected hip.

Exclusion criteria: ① patients with severe cardiopulmonary, hepatic and renal incompetence, or coagulation dysfunction. ② patients unable to undergo surgery within 3 weeks due to open injury, infection, or combined injury. ③ Combined pelvic fracture.

### General information

This retrospective clinical comparative study was approved by the Medical Ethics Committee of Honghui Hospital Affiliated to Xi’an Jiaotong University(No. 202,303,065), and all patients’ written informed consent was obtained. We searched the medical system records for patients with acetabular dome impaction fractures who were surgically treated and divided into X-ray and O-arm groups based on intraoperative fluoroscopy between October 2018 and October 2021. A total of 48 patients were included, including 25 in the X-ray group and 23 in the O-arm group.The preoperative general data of the patient, such as gender, age, injury mechanism, injury type, and compound injury, are shown in Table 1.

**Table 1:** Comparison of preoperative general data

	the X-ray group	The O-arm group	p
No.of patients	25	23	
Age ( (years)	62.16 ± 10.59	61.04 ± 12.64	0.741
Sex			
Male	17	16	0.907
Female	8	7	
Mechanism of injury			
Traffic accident injury	15	12	0.611
High falling	4	5	
Walking injury	6	6	
BMI (Kg/m^2^)	25.44 ± 1.96	25.35 ± 1.67	0.862
Combined injury			
Upper limb fracture	1	1	0.440
Lower limb fracture	2	1	
Rib fracture	1	0	
Judet-Leteurnel classifification			
Anterior column	1	1	0.817
Anterior column and posterior Hemitransverse	13	14	
Both column	7	5	
T type fracture	3	2	
Transverse fracture	1	0	
Posterior column and Posterior wall	0	1	
Approach			
Ilioinguinal	15	17	0.307
The lateral rectus	4	3	
Kocher-Langenbeck	3	1	
Ilioinguinal + Kocher-Langenbeck	1	1	
Stoppa	2	1	
Days from admission to operation (days)	4.72 ± 1.75	4.96 ± 1.40	0.609
Length of stay in hospital (days)	9.56 ± 2.43	9.91 ± 1.65	0.563

### Surgical procedures

#### Preoperative preparation

The external protective device was removed before operation, and imaging data such as standard positive oblique pelvis X-ray and CT scan were obtained under supracondylar traction. the type of fracture and the location of the compression fracture was accurately determined to avoid misjudgment of the severity of the injury [14, 15]. The patient was positioned in a supine position on a radiolucent traction table (Mizuho OSI)with traction applied to the injured limb with 45◦ of knee flexion (to decrease tension in the sciatic nerve). An O-arm imaging device (Medtronic, Navigation, Inc) was then introduced to check whether the device was operating in conflict with the patient or the operating table.

#### Intraoperative operating

According to the type of fracture, different surgical approaches were chosen. Femoral supracondylar traction was continued to eliminate the pressure of the femoral head on the acetabulum. Preferential reduction of the posterior column can provide column stability for the management of compression fractures. Before the reduction of the posterior column, if the compressed bone block hindered the reduction of the posterior column, a hemostat was used to pry the compressed bone block in the direction of the femoral head. After the space occupation was released, the posterior column was reduced with a top rod. The support plate was implanted with partial screw fixation, which can preserve a larger fracture gap for subsequent treatment of compressed bone fragments.The anterior wall fragment was opened anterolaterally or a window was opened from the anterior column of the acetabulum, and the location of the compressed fragment was carefully identified. The bone fragment was usually compressed into the cancellous bone behind and above the acetabulum, and it was generally difficult to find the fracture gap. Careful exploration with hemostatic forceps was required above the compressed bone fragment to find the section where the cancellous bone was compressed and softened. A flat-headed top rod or a periosteal ion was then used to reset the bone block, and the traction slightly relaxed to prevent the broken bone block from falling into the joint space. After the reduction was completed, the Kirschner wire was temporarily fixed.

The X-ray group:The reduction of the fracture was evaluated by X-ray fluoroscopy. After the arc Angle of the acetabulum was consistent with the top of the femoral head under multi-angle fluoroscopy, autologous bone or allograft bone was implanted in the bone defect area, and the fracture was fixed with plate screws.

The O-arm group: the fracture reduction was evaluated using the 2D fluoroscopy of the O-arm. If the consistency between the acetabulum and femoral head was found to be good under 2D fluoroscopy, the 3D scan of the O-arm was used for verification. After scanning, if a large step-off or gap shift in the acetabular dome was found, the Kirschner wire or screw in the scan was used as a reference to determine the specific position of the unreduced bone. Fragment in the acetabular dome and re-reduction was performed again.The final verification using an O-arm scan showed that the residual step-off and gaps of the acetabular dome were repositioned satisfactorily, and the consistency of the acetabulum and femoral head was good. The fracture was fixed with plate screws.

#### Postoperative management

Antibiotics were used to prevent infection After the operation, and the drainage tube was removed when the drainage volume was lower than 50 ml/24 h. Conventional CT scan was performed in the X-ray group, while no CT scan was required in the 0-arm group. The patient could sit up 2–3 weeks after surgery, and restricted weight bearing for 6 weeks after surgery, and then gradually carried out partial weight bearing until the 12th week.

### Observatian indicators

**Fig. 2:**
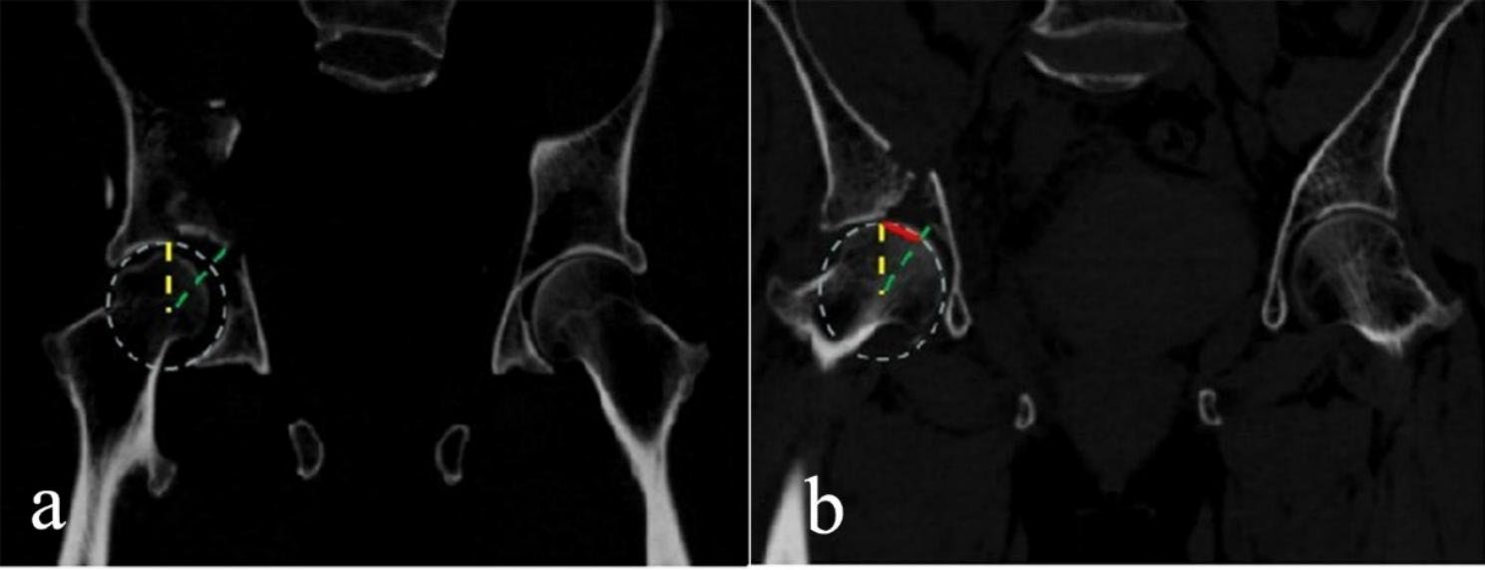
Measurement technique for the articular gap and step-off. A circle is drawn to match the articular surface. (**a**) The step-off is the highest point of the compression bone block and the circle (green and yellow lines). (**b**) The gap is the distance between the displaced and nondisplaced fracture end and the circular intersection (red line)

Gender, age, body mass index, injury cause, fracture type, combined injury and other general information of patients were recorded. The operative time, intraoperative blood loss and postoperative follow-up time were recorded. Fracture reduction was recorded.The results of surgical imaging scans were evaluated by two orthopedic surgeons not involved in the surgery in a case-randomized order. Acetabular dome residual step-off and gap displacement were measured under the PACS system using the technique described by Verbeek et al. [16] (Fig. 2), which has shown excellent interobserver and intraobserver reliability. The quality of fracture reduction was assessed according to the criteria described by Verbeek et al. [17]and Moed et al. [18]: anatomical reduction (0 < step-off ≤ 1 mm, 0 < gap ≤ 2 mm), good reduction (1 < step-off ≤ 2 mm, 2 < gap ≤ 10 mm), or poor reduction (step-off > 2 mm, gap > 10 mm). The postoperative functional recovery of the affected hip was recorded, and the patients were followed up regularly. At the last follow-up, hip function was evaluated according to the modified Merle d’Aubigné and Postel score [19].

### Statistical analysis

Statistical analysis was performed using SPSS version 20.0 (SPSS Inc., Chicago, IL, USA), and the Shapiro-Wilk test was used to determine whether the data were normally distributed. Independent sample t-test was used for comparison of numerical variables. Chi-square test was used to compare the classified data, and p < 0.05 was considered statistically significant.

## Results

**Fig. 3:**
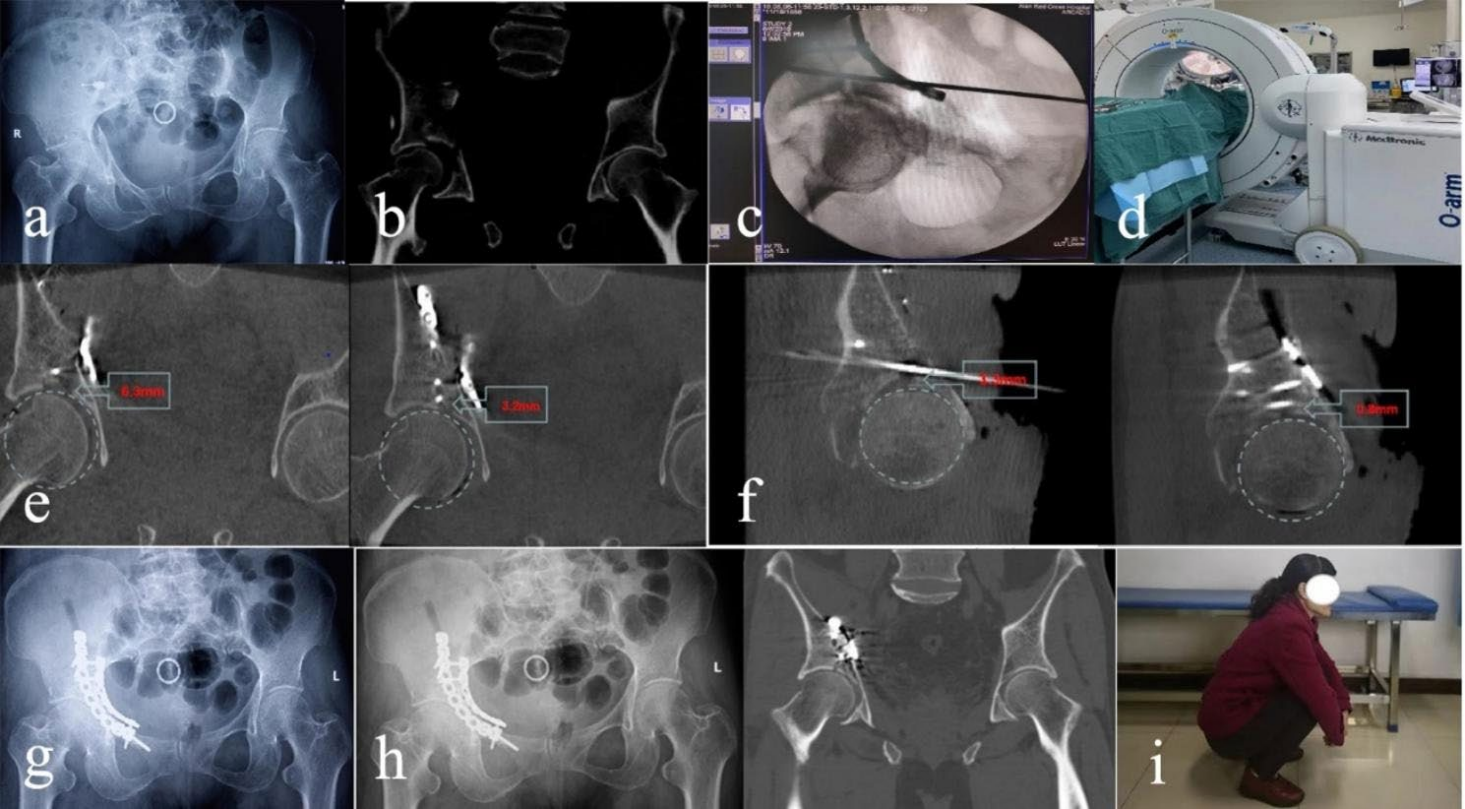
A female patient, aged 64, who presented with anterior column and posterior hemi-transverse fracture from a walking fall injury. (**a, b**) Preoperative X-ray and CT scan revealed acetabular dome impaction fractures. (**c**) X-ray showed good consistency of the head and socket after initial reduction and satisfactory reduction of the fracture. (**d**) Intraoperative O-arm scan was used to assess fracture reduction. (**e**) The residual gap displacement of the acetabular dome was reduced from 6.3 mm to 3.2 mm in the first and last O-arm scans. (**f**) The residual step-off displacement of the acetabular dome was reduced from 3.3 mm to 0.8 mm in the first and last O-arm scans. (**g**) Postoperative X-ray showed satisfactory reduction of the fracture. (**h**) X-ray and CT scans 11 months after surgery showed union. (**i**) The affected hip functioned well 11 months after surgery

There were no significant differences in preoperative general information, such as gender, age, injury type, combined injury, between the two groups (p > 0.05) (Table 1). The average operation time was171.68 ± 32.99 min and 205.52 ± 36.4 min in the X-ray group and the O-arm group.the average intraoperative blood loss was 966.80 ± 418.51 ml and 1293.91 ± 427.98 ml in the X-ray group and the O-arm group. The mean residual average step displacement in the acetabular dome region was 3.48 ± 2.43 mm and 1.61 ± 1.16 mm (p < 0.05), while the mean gap displacement was 6.72 ± 3.69 mm and 3.83 ± 1.67 mm (p < 0.05) in the X-ray and the O-arm groups, respectively. In the X-ray group, according to the fracture reduction criteria described by Verbeek and Moed et al., one case was excellent, 13 cases were good, 11 cases were poor; 56% were excellent or good. In the O-arm group, seven cases were excellent, 12 cases were good, and four cases were poor; overall in this group, 82.6% were excellent or good. The quality of postoperative fracture reduction was statistical differences between the two groups(p < 0.05). Operation time and intraoperative blood loss were increased in the O-arm group, and there were statistical differences between the two groups (p < 0.05). Two patients lost follow-up, 46 patients gained follow-up, fracture healing, no infection occurred after operation. In the X-ray group, according to the modified Merle d’Aubigné and Postel function score, three cases were excellent, 12 cases were good, six cases were middle, three cases were poor; 62.5% were excellent or good, In the O-arm group, 15 cases were excellent, four cases were good, two cases were middle, one case was poor; 86.4% were excellent or good. Postoperative hip function scores were statistically different between the two groups(p < 0.05) (Table 2). At the last follow-up, in the X-ray group, three patient developed osteoarthritis, one patient developed ectopic ossification, and two patient developed femur head necrosis. In the O-arm group, one patient developed osteoarthritis (p < 0.05). Typical cases are shown Fig. 3.

**Table 2:** Reduction quality and function follow-up results

	the X-ray group (n = 25)	The O-arm group (n = 23)	p
Operative time (mins)	171.68 ± 32.99	205.52 ± 36.41	0.001
Intraoperative blood loss(ml)	966.80 ± 418.51	1293.91 ± 427.98	0.01
Follow-up time (month)	24.08 ± 6.87	25.14 ± 7.69	0.626
Imaging assessment results			
Step-off (mm)	3.48 ± 2.43	1.61 ± 1.16	0.002
Gap(mm)	6.72 ± 3.69	3.83 ± 1.67	0.001
According to Verbeek,Moed et alAnatomical reduction			
Excellent	1	7	0.025
Good	13	12	
Poor	11	4	
The modified Merled’Aubigne & Postel scoring system			
Excellent	3	15	0.001
Good	12	4	
Middle	6	2	
Poor	3	1	
Complications			
Osteoarthritis	3	1	0.043
Ectopic ossification	1	0	
Femur head necrosis	2	0	

## Discussion

### Three-dimensional imaging scans assess importance of ADIF reduction

In recent years, great progress has been made in the treatment of ADIF, especially in terms of morphology, reduction and fixation techniques for compressed bone fragments [5, 6, 20–23]. However, surgical failure rates remain high and clinical outcomes are poor, especially in elderly patients with acetabular roof compression fractures. Compared with other types of acetabular fractures, ADIF features fracture lines on the anterior and posterior columns and has different forms of compressed bone fragments inside the acetabulum similar to type 2 fractures of the tibial plateau. However, the acetabulum has an articular surface with a spherical multi-arc structure. Therefore, during ADIF reduction, reduction of the anterior and posterior columns can be assessed by observing the continuity of the iliopubic and ilioischial lines using fluoroscopy in the anteroposterior and double oblique positions. However, in the reduction of compressed bone fragments, owing to the particularity of the location, the continuity of the top arc can only be observed through a two-dimensional image provided by X-rays, which is limited and requires a 3D imaging scan for observation [7, 24].

Several studies have shown that CT scans can provide more accurate and detailed information than radiographs in evaluating the quality of acetabular fracture reduction [9, 18, 25, 26]. Moed et al. [18] performed postoperative CT in 67 patients with acetabular posterior wall fractures. According to the X-ray images, the quality of reduction in 65 cases was anatomical reduction, and in two cases was incomplete reduction. However, postoperative CT scans showed 11 cases with a step shift greater than 2 mm and 24 cases with a gap shift greater than 1 cm. Meesters et al. [26] measured the step and space displacement in postoperative X-ray and CT scans of patients with acetabular fractures and re-evaluated the intraoperative imaging results. They found that compared with postoperative CT, the X-ray images missed 52% of the gap and 80% of the step displacement, and intraoperative X-ray images missed approximately 70% of the residual gap and step displacement. Although previous studies have shown that CT has certain advantages in evaluating the quality of acetabular fracture reduction, owing to the poor mobility of conventional CT equipment and the increased radiation dose to doctors and patients, no scholars currently use conventional CT to evaluate fracture reduction during surgery. Instead, CT was performed after acetabular fracture surgery [27].

### Advantages and disadvantages of O-arm evaluation of reduction quality in ADIF

With the development of medical technology, the O-arm has been used in acetabular fracture surgery because of its advantages, such as instant 3D image generation and good mobility. Sebaaly et al. [28] reported that use of an O-arm combined with a navigation system to treat acetabular fractures improved the accuracy of articular surface reduction and screw placement. However, their study analysed different types of acetabular fractures rather than a single type of fracture; therefore, the intraoperative application value of the O-arm could not be specifically determined. Rizkallah et al. [29], in their study on the clinical impact of intraoperative conical beam tomography and navigation on displaced acetabular fractures, showed that the reduction quality of acetabular fractures was significantly improved, and O-arm combined navigation therapy could significantly reduce the need for postoperative total hip replacement in patients with preoperative compression fractures. This is similar to the satisfactory results achieved in the postoperative fracture reduction and functional rehabilitation in this study.

In our study, the O-arm had the following advantages: (1) Significantly improvement in the quality of fracture reduction: Using the screw and gram needle in the O-arm scan as a reference to determine the specific position of the poorly reduced bone fragment and determining the specific reduction direction, the displacement direction of the compressed bone fragment is usually backward and upward compression, and the reduction direction should be anterior and downward. It is inappropriate to perform a single downward reduction of the acetabulum with an upward elevation of the top arc of the acetabulum based only on radiographic fluoroscopy. In this study, 23 ADIF cases were evaluated using the O-arm, with a good-to-excellent reduction rate of 82.6%. (2) Ensuring the safety and effectiveness of the operation: During the operation, screw position can be clearly identified by the O-arm scanning, and the screw can be removed in time if the position is poor. Accurate reduction of the acetabular dome area provides a good conditions for hip joint functional recovery in the later stages and improves the clinical efficacy of surgery. In this study, the excellent and good rate of modified Merled ‘Aubigne &Postel functional score reached 86.4% in 22 patients in O-arm group at the last follow-up. (3) Reduced the pollution of the operation area: During the whole operation, due to the protective effect of the “O”-shaped shell and the X-ray tube, the X-ray receiver will automatically rotate to the corresponding operation site and eliminate the need to manually adjust the position of the machine, so the sterile area in the operation is well protected, thereby reducing pollution of the surgical area. (4) Reduced overall radiation dose: The radiation dose used by a single O-arm during an operation is equivalent to 60% of that of ordinary CT [30], and a 3D scan of the O-arm is used in the evaluation after satisfactory reduction of in ADIF. Moreover, two-dimensional fluoroscopy was used to assess the quality of reduction when the initial reduction was completed, and postoperative CT was not required.

However, application of the O-arm has limitations: (1) Increased operation time and intraoperative blood loss: Owing to its large size, slow moving speed, and relatively complicated operation, the O-arm increases the operation time and the amount of intraoperative blood loss; and (2) Increased healthcare costs: O-arm equipment is expensive, and areas with relatively backward economies cannot enjoy high-quality medical resources.

### Limitations

This study had multiple limitations. The number of samples included was small, the types of ADIF were incomplete, and this was a single-centre study. Its follow-up time was short; therefore the long-term efficacy could not be further evaluated. Additionally, the O-arm has only recently been used in the treatment of acetabular fractures, and the complexity of the operation and low proficiency of imaging personnel led to a long operation time, which may have affected our results.

## Conclusions

The application of O-arm in ADIF can have a positive impact on the improvement of fracture reduction quality and functional recovery, and has high application value.

## Data Availability

The datasets used and/or analyzed during the current study are available from the corresponding author on reasonable request.

## List of abbreviations

ADIF: Acetabular dome impaction fracture
BMI: body mass index
3D: three-dimensional
O-arm: intraoperative mobile 2D/3DX imaging system
